# Development of a Dual MOS Electronic Nose/Camera System for Improving Fruit Ripeness Classification

**DOI:** 10.3390/s18103256

**Published:** 2018-09-27

**Authors:** Li-Ying Chen, Cheng-Chun Wu, Ting-I. Chou, Shih-Wen Chiu, Kea-Tiong Tang

**Affiliations:** Department of Electrical Engineering, National Tsing Hua University/No. 101, Sec. 2, Kuang-Fu Road, Hsinchu 30013, Taiwan; cly901213@gmail.com (L.-Y.C.); clarence1222@gmail.com (C.-C.W.); billy22109@gmail.com (T.-I.C.); swchiu1984@gmail.com (S.-W.C.)

**Keywords:** Electronic nose (E-nose), volatile organic compounds (VOCs), fruit odor, maturity

## Abstract

Electronic nose (E-nose) systems have become popular in food and fruit quality evaluation because of their rapid and repeatable availability and robustness. In this paper, we propose an E-nose system that has potential as a non-destructive system for monitoring variation in the volatile organic compounds produced by fruit during the maturing process. In addition to the E-nose system, we also propose a camera system to monitor the peel color of fruit as another feature for identification. By incorporating E-nose and camera systems together, we propose a non-destructive solution for fruit maturity monitoring. The dual E-nose/camera system presents the best Fisher class separability measure and shows a perfect classification of the four maturity stages of a banana: Unripe, half-ripe, fully ripe, and overripe.

## 1. Introduction

Odor sensing technology [1,2,3] has been widely used, such as in indoor air quality monitoring, medical care, public security, food freshness control, environmental quality monitoring, military applications, and hazardous gas detection. In agriculture, fruits generate different volatile organic compounds (VOCs) [4] of varying concentrations in different stages of maturity. This is a commonly used indication of fruit maturity based on the biological mechanism. In most areas where agriculture [5] is the primary economic activity, farmers still evaluate the maturity and quality of their crops through experience, which is not sufficiently efficient and is unscientific. Therefore, developing a scientific method of monitoring fruit maturity has become a crucial topic in modern agriculture. Electronic nose (E-nose) systems [6,7,8,9,10,11] incorporating a camera has been proposed to monitor fruit maturity. The E-nose system mimics human olfaction to detect odors using a gas sensor array. The array comprises different gas sensors and detects the VOCs emitted by fruits. In addition to detecting the VOCs, camera systems have also been used to monitor fruit color changes.

Unlike most of the traditional methods of assessing fruit ripeness by measuring firmness, pH, and sugars contents, the methods of using smell (E-nose) [12,13,14,15] and camera (computer vision) [16,17,18,19] are non-destructive, and thus more desirable. Each fruit has its own characteristic odor, which constitutes the VOCs concentrations. The VOCs composition continuously changes in post-harvest fruit during the maturation process. The color of the fruit peel is also a good indicator of different ripeness stages. The color can be decomposed into red-green-blue (RGB) as three coordinates. The RGB composition also continuously changes during the maturation process.

This study proposes a dual E-nose/camera system that utilizes both smell and vision information for improving fruit ripeness classification. This non-destructive system aims to monitor fruit maturity and provide better accuracy in identifying fruit ripeness stages.

## 2. Materials and Methods

We purchased the bananas from a local market. We used a bunch of bananas weighing about 1.2 kg. The cultivar of the bananas was *Musa basjoo Siebold var. formosana*. All the bananas were purchased when the peel color was green (unripe). Harvested unripe bananas were stored in a large sample chamber to prevent contamination from external odors. All the experiments were controlled at 20 °C–25 °C and a relative humidity of 70–80% under normal storage conditions. The color changes of the peel were recognized as the maturity states in the experiment, namely, unripe, half-ripe, fully ripe, and overripe. During the four maturity stages of the bananas, this study was carried in relation to three aspects. First, a thermal desorption-gas chromatography-mass spectrometry (TD-GC-MS) system was employed to analyze the odor composition of volatile organic compounds (VOCs). Second, the proposed E-nose system was used to record and analyze the data. Third, a camera system was employed to take pictures of the bananas for further analysis.

### 2.1. The Maturity Stage Determination

Bananas are one of the most important fruit merchandises. Bananas are usually harvested at the unripe stage (green) and they remain green and firm without significant changes in peel color and texture before ripening [20]. During the ripening time, the banana peel color changes, the flavor develops, and the fruit softens. The first observable sign is the peel color changing from green to yellow. The traditional method to grade the matured stages of bananas is through eye inspection of changes in the peel color, as shown in Table 1 [21]. According to the table, we defined the maturity of the bananas according to four stages: unripe, half-ripe, fully ripe, and overripe, corresponding to indexes 1, 2–5, 6, and 7, respectively. The duration of the peel color turning from green to yellow is defined as the half-ripe stage (index 2–5).

### 2.2. TD-GC-MS System

The volatile organic compounds (VOCs) composition of the bananas was analyzed using a thermal desorption-gas chromatography-mass spectrometry (TD-GC-MS) system. Stainless steel tubes (6 mm × 5 mm × 90 mm) filled with sorbents (200 mg Tenax TA (60/80 mesh)) were used as traps for the sample collection with simultaneous preconcentration for 1 h. The sampled analytes were released from the sorbents through thermal desorption. The flow rate of the carrier gas through the sorption trap during desorption was 100 mL/min. The initial temperature was increased to 200 °C and then held for 10 min. Liquid nitrogen was used for cryofocusing the desorbed analytes at −20 °C. For subsequent sample injection into the capillary, the column was heated at a rate of 99 °C/s up to 200 °C.

The analysis was conducted on a 7890 A gas chromatograph equipped with a mass selective detector 5973 C (both from Agilent Technologies, USA) with sample injection by means of thermal desorption, as described in the previous paragraph. A capillary column (30 m × 0.25 mm × 0.25 μm, J & W Scientific) was used. The oven temperature was programmed as follows: initial 35 °C (held for 6 min), then ramped 3 °C/min up to 135 °C (held for 5 min), then again ramped 6 °C/min to 230 °C (held for 5 min). The MS analyses were performed in a Total Ion Chromatogram (TIC) mode, with a scan range of 35 to 500 amu. Ionization of the separated compounds was carried out through electron impact ionization at 70 eV. The chromatographic data were acquired using the Agilent Chemstation Software (GC-MS Data Analysis from Agilent). The mass spectrum library NIST08 was applied for the identification of the detected compounds. The match quality value of the compounds we approved had to be over 70.

The TD-GC-MS [22] system was used to analyze and identify the composition of VOCs emitted by the fruit at different stages of maturity using a compatible database. Table 2 presents the TD-GC-MS analysis of the spectrum results. This study identified the complex odors of the fruits by using the proposed system.

### 2.3. E-Nose System

**Figure 1:**
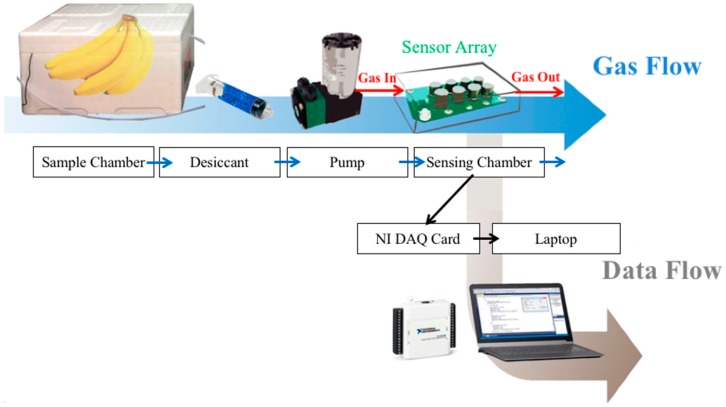
Block diagram of the proposed electronic nose (E-nose) system.

Figure 1 shows the block diagram of the proposed electronic nose (E-nose) system. The fruit samples were kept in the sample chamber. To prevent the influence of moisture, odors were passed through the desiccant before being pumped into the sensing chamber where the sensor array was inside. The sensor responses were acquired using a National Instrument data acquisition card (DAQ card, USB-6009, National Instruments Corporation, Austin, TX, USA) and stored on a laptop. The desiccant consisted of silica gel and cobalt chloride. Polytetrafluoroethene (PTFE) was used for the pneumatic circuit to avoid odor attachment.

The gas measurement process was divided into two phases, namely, collection and desorption. During the collection phase, the odor of the sample was pumped into the sensing chamber. Data were collected for 5–10 min. This collection period was sufficient for the sensor resistances to reach a steady state. After the sample collection was complete, the odor was eliminated through desorption. During the desorption phase, clean dry air was passed through the sensing chamber for a moment to ensure that the sensors reached their baseline. To obtain numerous data samples, the experiment collection cycle was repeated. The entire process was controlled using a LabVIEW program installed on the laptop.

#### 2.3.1. Sample and Sensing Chambers

**Figure 2:**
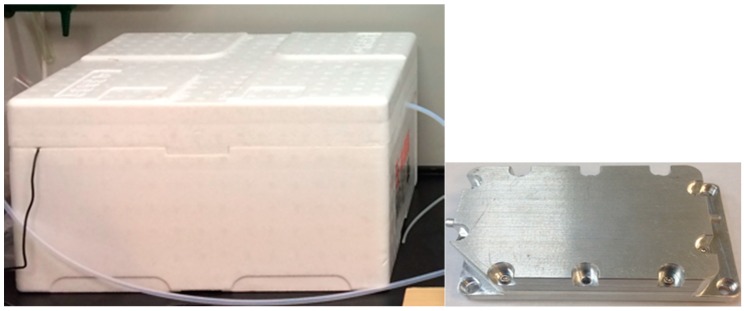
Sample chamber (**left**) and sensing chamber (**right**).

The object under testing was kept in the sample chamber, while the sensor array was placed in the sensing chamber, as shown in Figure 2, respectively. The sample chamber was a Styrofoam box and had a volume of approximately 5000 cm^3^. The sensing chamber was made of metal (stainless steel) to avoid odor attachment and had an inner volume of approximately 15 cm^3^.

#### 2.3.2. Sensor Array

The sensor array was constructed using seven MOS gas sensors from FIGARO Engineering Inc.: TGS2600, TGS2602, TGS2603, TGS2610, TGS2611, TGS2612, and TGS2620 [23,24,25,26,27]. Table 3 indicates that each sensor was labelled from S1 to S7 and summarizes the target gases of each sensor. The resistivity of the sensors increased in the presence of air and decreased in the presence of target VOCs or odors.

#### 2.3.3. Data Acquisition and Feature Extraction

A data acquisition (DAQ) card was used for data acquisition in this study. The DAQ was controlled using LabVIEW on a laptop. The sensor responses were stored on the laptop using the DAQ, and these data sets were analyzed to extract information. Once the sensor responses reached a steady state, the sensor resistance change ratio (ΔR/R) was extracted as features for classification.

### 2.4. Camera System

Pictures of the bananas were captured using a digital camera (HERO+, GOPRO Inc., San Mateo, CA, USA) and analyzed using ImageJ. The red-green-blue (RGB) values were obtained from fruit images, and color characteristics were calculated according to the pixel value (0–255) of each color component. Signal preprocessing was employed to extract the relevant data from these features and prepare the data for a multivariate pattern analysis.

**Figure 3:**
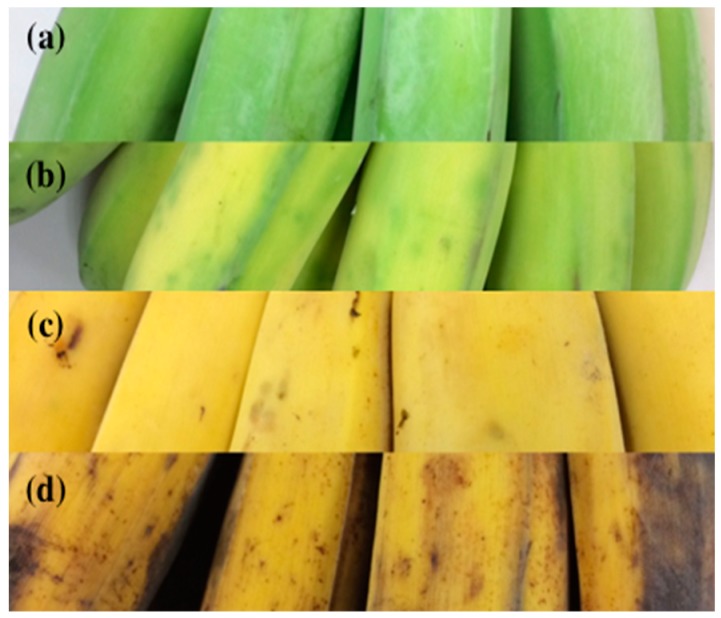
Banana samples in different maturity states: (**a**) unripe (all green); (**b**) half-ripe (green with yellow); (**c**) fully ripe (full yellow); and (**d**) overripe (yellow with brown).

Figure 3 presents pictures of bananas in the four maturity stages in this study. The color of the bananas significantly changed during the ripening process, and this information can be used to investigate the maturity of bananas. The color changes in the bananas were observed daily using a camera. According to the color variation, the maturity of the bananas was defined as follows: unripe: all green; half-ripe: green with yellow; fully ripe: yellow; and overripe: yellow with brown.

## 3. Results and Discussion

### 3.1. Sensor Response and Color Measurement

The sensors were preheated for 30–60 min before beginning the experiment. The experiment was performed 15 times each day, providing 105 measurements in a total of 7 days. Banana ripening was completed within seven days. According to the peel color changes of the banana, the daily measurements were divided into four groups as follows: unripe (day 1); half-ripe (day 2 and day 3); fully ripe (day 4, day 5, and day 6); and overripe (day 7). As a result, there were 15 unripe samples, 30 half-ripe samples, 45 fully ripe samples, and 15 overripe samples.

**Figure 4:**
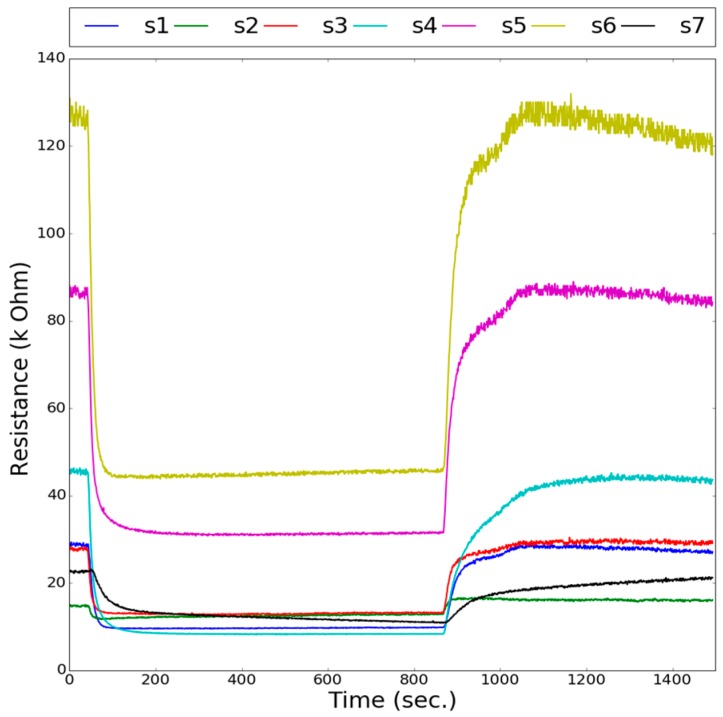
A typical sensor response: S1 to S7 are sensor labels, as listed in Table 3.

**Figure 5:**
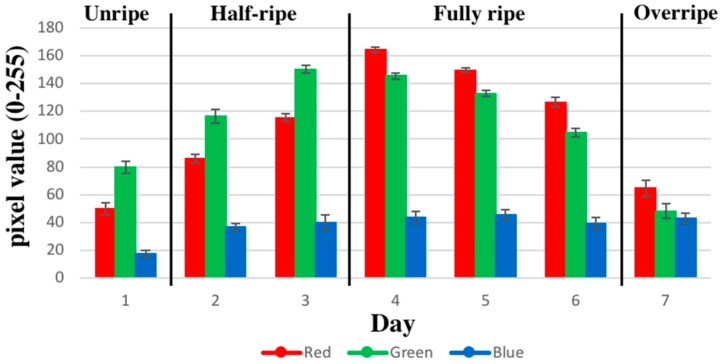
The daily red-green-blue (RGB) measurements.

Figure 4 shows a typical sensor resistance response. The sensor resistance change ratio (ΔR/R) was extracted as features for further classification. The pictures of the banana were taken 15 times each day. The variation in the color of the bananas in terms of RGB was recorded as a pixel value (0–255), as shown in Figure 5.

For classification, each data sample set comprised the seven sensor responses and RGB values of the fruit surface color. The classification algorithms were performed with the leave-one-out validation method. The data analysis and classifier algorithm were developed using the Python machine learning library.

### 3.2. Classification with E-Nose System

**Figure 6:**
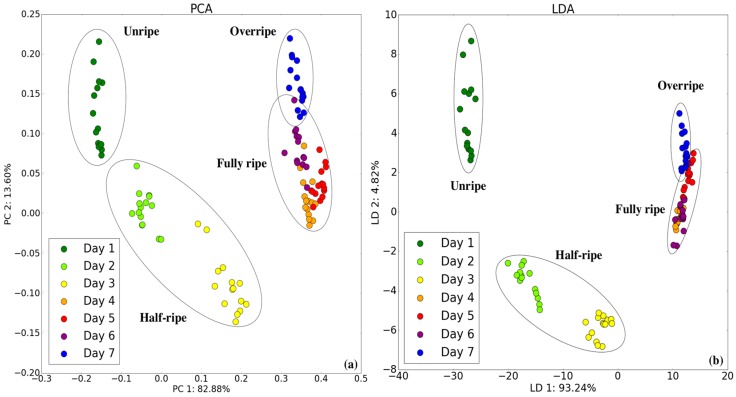
Two-dimensional (**a**) principal component analysis (PCA) and (**b**) linear discriminant analysis (LDA) plots by E-nose in all maturity states.

Principal component analysis (PCA) [13] and linear discriminant analysis (LDA) [28] were applied to the 105 measurements obtained using the E-nose system to determine whether the sensor array could distinguish between different ripening states. The input values were normalized prior to the PCA and LDA. Figure 6 displays the PCA and LDA results on a two-dimensional plane. According to the PCA and LDA, unripe and half-ripe are clustered from the left to the middle of the plot, whereas fully ripe and overripe are located on the right side of the plot. While showing a clear boundary between unripe and half-ripe, the figure indicates a slight overlap between fully ripe and overripe.

### 3.3. Classification with Camera System

**Figure 7:**
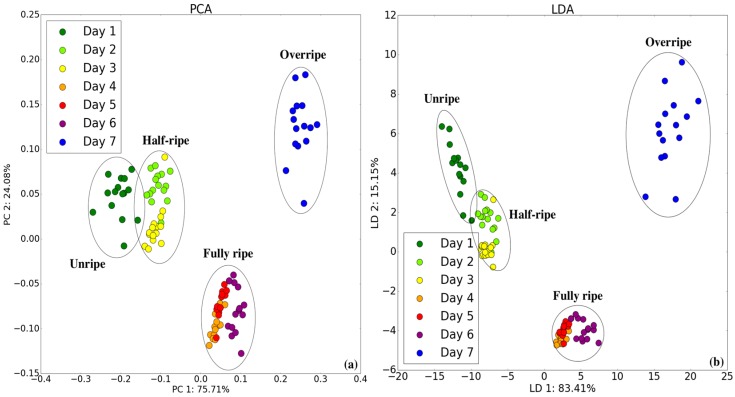
Two-dimensional (**a**) PCA and (**b**) LDA plots by camera in all maturity states.

RGB can be used to describe the amount of red, green, and blue color components forming a color feature. Figure 7 displays the PCA and LDA results on a two-dimensional plane using RGB. According to the PCA and LDA, unripe and half-ripe are located on the left of the plot, whereas fully ripe is located at the bottom, and overripe is located at the right. Clear boundaries can be found to distinguish the four stages, while unripe and half-ripe are close in the plot.

### 3.4. Classification with the Camera/E-Nose System

**Figure 8:**
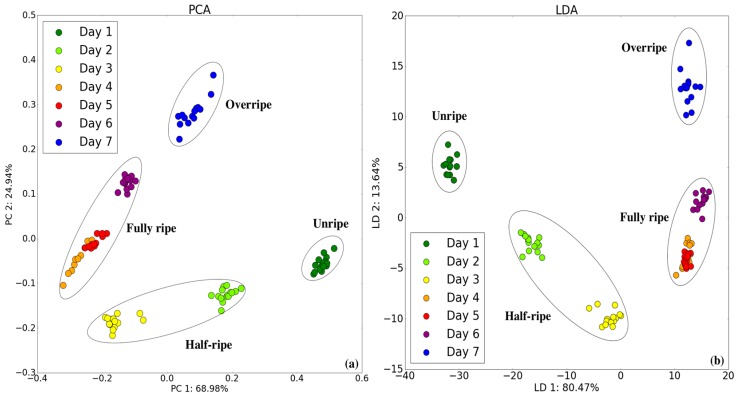
Two-dimensional (**a**) PCA and (**b**) LDA plots of E-nose/camera system in all maturity states

More comprehensive data were acquired and a multivariate pattern could be analyzed by adding a color feature to the original responses of the seven sensors. Each new data point comprised the resistance change ratio (ΔR/R) values from the seven sensor responses and three RGB values. Figure 8 shows new PCA and LDA score plots for the bananas in all maturity states. The figure shows that all the groups are completely separate from each other. The Fisher class separability measure [29] shows a significant improvement from 6.93 (E-nose only) and 8.08 (Camera only) to 10.52 (Camera/E-nose), as shown in Table 4. The results obtained using the PCA and LDA plot for the ripening period indicate a perfect classification.

We further applied K-nearest neighbor (KNN) [30,31] and support vector machine (SVM) [31] for discrimination [25,32]. Table 5 shows the classification results using the leave-one-out cross validation method. While the E-nose system or camera system alone provide a reasonably good classification, incorporating these two systems results in even better accuracy. Table 6 presents the actual data showing the accuracy of the ripeness determinations using the E-nose and camera system for each of the four sample types tested. Table 7 presents a comparison of this study and prior studies using the E-nose or camera system for fruit ripeness classification. References [12,13,14] used the E-nose system to classify bananas and pears, resulting in 83.4–98.66% accuracy. References [16,17,18] used computer vision to classify bananas, limes, and pineapples, resulting in 75–99% accuracy. The proposed dual E-nose/camera system exhibits a superior performance than using only either an E-nose or a computer vision system.

## 4. Conclusions

We proposed three methods to monitor the four maturity stages of bananas. For the four stages (unripe, half-ripe, fully ripe, overripe) during a seven-day study, both the E-nose system and the camera system exhibited good potential to identify the four stages. By incorporating the E-nose and camera together, the dual system demonstrated perfect classification results. This proposed dual E-nose/camera system provides a non-destructive solution for fruit maturity monitoring. The proposed system is very suitable for the prediction of shelf-life in supermarkets.

## Figures and Tables

**Table 1:** Color index of banana fruits in various scales.

Index.	Color	Stage
1	All green	Unripe
2	Green with a trace of yellow	Half-ripe
3	More green than yellow	
4	More yellow than green	
5	Yellow with green necks	
6	All yellow	Fully ripe
7	All yellow with brown	Overripe

**Table 2:** Typical volatile organic compounds (VOC) composition results in banana fruit at each stage of maturity.

Volatile Organic Compounds.	Unripe	Half-Ripe	Fully Ripe	Overripe
**Alkanes**				
Isobutane	*	*	*	*
Butane	*	*	*	*
Pentane	*	*	*	*
Cyclopentane	*	*		
2-Pentanone		*	*	*
1,3-Butadiene, 2-methyl-			*	*
Cyclobutane, methyl-			*	
Bicyclo[4.2.0]octa-1,3,5-triene				*
**Alcohols**				
Ethyl alcohol		*	*	*
1-Propanol, 2-methyl-		*	*	*
1-Butanol		*	*	
1-Butanol, 3-methyl-		*	*	*
2-Pentanol			*	*
**Esters**				
Formic acid, ethyl ester				*
Ethyl Acetate		*	*	*
n-Propyl acetate		*	*	*
Acetic acid, 2-methylpropyl ester		*	*	*
Butanoic acid, ethyl ester		*	*	*
Acetic acid, butyl ester		*	*	
2-Pentanol, acetate		*	*	*
1-Butanol, 3-methyl-, acetate		*	*	*
Butanoic acid, butyl ester			*	*
Butanoic acid, 1-methylbutyl ester				
Butanoic acid, 2-methylpropyl ester		*	*	*
Butanoic acid, 3-methyl-, 2-methylpropyl ester		*	*	*
Butanoic acid, 3-methyl-, butyl ester		*	*	
Butanoic acid, 3-methyl-, 3-methylbutyl ester		*	*	*

**Table 3:** Figaro gas sensors used in the E-nose design and the target gases or VOCs to which the sensors are sensitive.

Sensor Number.	Sensor Type	Target Gas (According to FIGARO® Datasheet)
1	TGS2600	Hydrogen, Carbon monoxide
2	TGS2602	Ammonia, Hydrogen sulfide
3	TGS2603	Trimethylamine, Methyl mercaptan
4	TGS2610	Butane, LP gas
5	TGS2611	Methane, Natural Gas
6	TGS2612	Methane, Propane, Iso-butane
7	TGS2620	Alcohol, Solvent vapors

**Table 4:** The Fisher class separability measurement scores.

Fisher Class Separability Measure.	
Feature Type	Score
Camera	8.08
E-nose	6.93
E-nose/Camera	10.52

**Table 5:** Classification accuracy for the three proposed systems (camera, E-nose, E-nose/camera).

System.	Number of Feature	PCA + KNN(K = 3)	PCA + SVM	LDA + KNN(K=3)	LDA + SVM
Camera	3	99.05%	97.14%	99.05%	94.29%
E-nose	7	98.10%	95.24%	90.48%	86.67%
E-nose/Camera	10	100%	100%	100%	100%

**Table 6:** The actual data showing the accuracy of ripeness determinations using the E-nose and camera system for each of the four sample types tested.

		Incorrect Classification				Correct Classification			
System	Algorithm	Number of Test Samples Incorrectly Classified/Total Number of Test Samples				Number of Test Samples Correctly Classified/Total Number of Test Samples			
		Unripe	Half-Ripe	Fully Ripe	Over-Ripe	Unripe	Half-Ripe	Fully Ripe	Over-Ripe
Camera	PCA + KNN(K = 3)	1/15	0/30	0/45	0/15	14/15	30/30	45/45	15/15
	PCA + SVM	3/15	0/30	0/45	0/15	13/15	30/30	45/45	15/15
	LDA + KNN(K = 3)	1/15	0/30	0/45	0/15	14/15	30/30	45/45	15/15
	LDA + SVM	3/15	3/30	0/45	0/15	12/15	27/30	45/45	15/15
E-nose	PCA + KNN(K = 3)	0/15	0/30	2/45	0/15	15/15	30/30	43/45	15/15
	PCA + SVM	0/15	1/30	2/45	2/15	15/15	29/30	43/45	13/15
	LDA + KNN(K = 3)	0/15	0/30	6/45	4/15	15/15	30/30	39/45	11/15
	LDA + SVM	0/15	0/30	3/45	11/15	15/15	30/30	42/45	4/15
E-nose/Camera	PCA + KNN(K = 3)	0/15	0/30	0/45	0/15	15/15	30/30	45/45	15/15
	PCA + SVM	0/15	0/30	0/45	0/15	15/15	30/30	45/45	15/15
	LDA + KNN(K = 3)	0/15	0/30	0/45	0/15	15/15	30/30	45/45	15/15
	LDA + SVM	0/15	0/30	0/45	0/15	15/15	30/30	45/45	15/15

**Table 7:** Comparison of this study with other E-nose or computer vision studies.

Application.	Category	Feature Attribute Measured	Accuracy	Reference
E-nose	banana	aroma	83.4–92%	[12]
	banana	aroma	98.66%	[13]
	pear	aroma	94.6%	[14]
Computer vision	banana	color	95.5%	[16]
	lime	color	99%	[17]
	pineapple	color	75%	[18]
Proposed method	banana	aroma, color	100%	
